# SDF2L1 Inhibits Cell Proliferation, Migration, and Invasion in Nasopharyngeal Carcinoma

**DOI:** 10.1155/2020/1970936

**Published:** 2020-08-07

**Authors:** Liqian Zhang, Zunni Zhang, Liuqun Qin, Xiang Shi, Qisheng Su, Wuning Mo

**Affiliations:** Department of Clinical Laboratory, First Affiliated Hospital of Guangxi Medical University, Nanning, Guangxi Zhuang Autonomous Region, China

## Abstract

The purpose of this study was to explore the relationship between stromal cell-derived factor 2-like 1 (SDF2L1) and nasopharyngeal carcinoma (NPC). 12 NPC tissues and 12 chronic nasopharyngitis tissues were involved in our study. Quantitative real-time PCR (qRT-PCR) and Western Blot were utilized to detect the expression of SDF2L1. Besides, immunofluorescence analysis was utilized to determine the protein expression of 97 paraffin-embedded NPC tissues and 58 nasopharyngitis tissues. Biological functional experiment included Cell Counting Kit-8 (CCK-8) assay, cell clone formation assay, cell scratch migration assay, Transwell migration assay, and Transwell invasion assay. All data were analyzed by SPSS. Results showed that downexpression of SDF2L1 was prominently present in NPC tissues and cells. Furthermore, silencing the expression of SDF2L1 promoted NPC proliferation, migration, and invasion in vitro, while overexpression of SDF2L1 has the opposite effect. In conclusion, SDF2L1 may act as a cancer suppressor gene, play a crucial role in the occurrence and development of NPC, and be a new therapeutic target or prognostic indicator for NPC.

## 1. Introduction

Nasopharyngeal carcinoma (NPC) is one of the commonest head and neck malignant tumors which have obvious differences in race, gender, and geographical distribution, [1–3] Southeast Asia has the highest incidence [4]. Globally, there are 87,000 cases of NPC every year, accounting for 0.6% of cancer deaths, approximately [5]. Therefore, it is necessary to find molecular markers for the early diagnosis of NPC and effective gene targets for treatment.

Stromal cell-derived factor 2-like 1(SDF2L1) is a member of stromal-derived factor family (SDF) and located on the human chromosome 22q11.21. In addition, SDF2L1 is a new inducing gene of endoplasmic reticulum stress. With the deepening of the study on SDF2L1, researchers found that SDF2L1 was located in the endoplasmic reticulum and not only participated in the endoplasmic reticulum stress but also regulated metabolism. Recently, studies reported that SDF2L1 may act as a tumor suppressor gene and play an important role in a variety of cancers, such as breast cancer and ovarian cancer [6, 7]. Nevertheless, the biological function of SDF2L1 in NPC still remains unknown.

Thus, the purpose of this study was to investigate the biological behavior of SDF2L1 in NPC by silencing and overexpressing the levels of SDF2L1 in NPC cells. Our research directly indicated that SDF2L1 as a tumor suppressor gene may play an important role in NPC occurrence and development.

## 2. Materials and Method

### 2.1. Cell Culture

The human nasopharyngeal carcinoma cell lines (HONE1, 5-8F) and the human normal nasopharyngeal epithelial cells lines (NP69) were obtained from the Otolaryngology laboratory of Guangxi Medical University (Nanning, Guangxi, China). These cells were cultured in a complete cell culture medium which contains 90% RPMI-1640, 10% fetal bovine serum (FBS), and the mixture of 100 *μ*g/ml penicillin-streptomycin and 100 *μ*g/ml amphotericin B at 37°C and 5% CO_2_ in a humidified incubator.

### 2.2. Clinical Samples

From March 2016 to June 2016, we collected 12 NPC tissues (male/female = 9 : 3, age: 56 (27~65), 12 nasopharyngitis tissues (male/female = 8 : 4, age: 54 (31~63), and 97 paraffin-embedded NPC tissues and 58 nasopharyngitis tissues for immunohistochemistry assay. All of the tissues were obtained from the First Affiliated Hospital of Guangxi Medical University, and the patients were confirmed by the pathology department and had no other tumors and patients with nasopharyngeal carcinoma were collected without chemotherapy and radiotherapy at the same time. Tumor staging was performed by at least 2 senior pathologists strictly following the TNM (tumor node metastasis) staging method of UICC and AJCC 2008. All patients in this study had signed informed consent forms, and we obtained the consent of the ethics committee of the First Affiliated Hospital of Guangxi Medical University.

### 2.3. Cell Transfection

Lentivirus silenced the expression of HONE1 and 5-8F (LV-SDF2L1-RNAi) and the control vector (LV-CON077), meanwhile, lentivirus overexpressed HONE1 and 5-8F (LV-SDF2L1) and the control vector (LV-CON238). All of these were bought from GeneChem (Shanghai, China). According to the manufacturers' instruction, we found that the best conditions for infection were when the inoculated cell density was 2.5 × 104/ml and the MOI of lentivirus infection to cells was 80. We inoculated cells into 6-well plates, and then according to cell MOI and virus titer, virus quantity was added for infection; after 72 h transfection, the transfection efficiency of cells was observed under the inverted fluorescence microscope (Olympus, Japan). The fluorescence efficiency over 80% can be used for the subsequent experiments. The successful transfection of HONE1 and 5-8F was determined by qRT-PCR.

### 2.4. Quantitative Real-Time PCR (qRT-PCR)

The total RNA of NPC tissues and cells were obtained according to the RNA extraction instructions of the TRIzol Reagent (Takara). For the synthesis of cDNA, PrimeScript™ RT reagent Kit with gDNA Eraser (Takara) was used. The operation of 7500 Real-Time PCR System was applied to verify the expression mRNA, using the SYBR Green PCR Master Mix (Takara). The primer sequence of qRT-PCR reaction were as follows: SDF2L1-F: TGTTCCTGTCAGTCACGGGT; SDF2L1-R: TGGCCTTCCACGTATTGTGC; *β*-actin-F: ACATCCGCAAAGACCTGTAC; and *β*-actin-R: ACATCCGCAAAGACCTGTAC. Fold change (relative expression) = 2^−ΔΔCt^.

### 2.5. Western Blot

Cells were cleaved in cell lysates containing 1% protease inhibitor (PSMF) which contains 1 ml RIPA and 1 *μ*l PSMF. The cell proteins were electrophoresed and transferred to a PVDF membrane using 12% isolated gel and 5% concentrated gel as carriers. Therewith, after blocking with 5% skim milk at room temperature for 1 h, these PVDF membranes were incubated with the SDF2L1-specific antibody and anti-*β*-actin (Aviva Systems Biology) at 4°C for at least eight hours, respectively. These PVDF membranes were then incubated with horseradish peroxidase-labeled secondary antibodies at room temperature for 1 h. Odyssey imaging system (LI-COR, US) was used to detect the protein expression on the PVDF membrane.

### 2.6. Immunohistochemistry Assay

After dewaxing, dehydration, and high-pressure antigen repair, paraffin sections were incubated in 3% hydrogen peroxide solution at room temperature for 20 min and then sealed in 5% normal goat serum (BSA) for 10 min. Next, the paraffin sections were washed with PBS for 3 times and then cultured in biotin-labeled secondary antibodies for 15 min. Besides that, the second-generation horserade-labeled streptomycin solution was added and incubated for 15 min. Then, the prepared DAB color reagent was added to the slice by drop and stained by hematoxylin for 1 min. The results were determined by the product of chromatin intensity scores and quantitative scores.

### 2.7. Cell Proliferation Assays

The Cell Counting Kit-8 (CCK-8) assay was carried out according to the instruction. The cells were diluted to 2.5 × 102/ml and inoculated in 96-well plates; each group of cells was parallel to 5 wells, and the assay was repeated for 3 times. After adding the CCK-8 reagent, the optical density (OD) value at 0 h, 24 h, 48 h, 72 h in 450 nm absorbance was detected.

As for cell clone formation assay and inoculation of 2 ml/well cell suspension into 6-well plates (HONE1: 400/ml, 5-8F: 500/ml), fresh medium was replaced every 3 d and cultured until clones were obviously visible. Thereafter, 0.1% crystal violet and 4% paraformaldehyde to fix and stain the clones were used.

### 2.8. Cell Migration and Invasion Assays

Briefly, for cell scratch migration assay, the prepared cell suspension was inoculated into the 6-well plate and incubated in the incubator with 37°C, 5%CO_2_. When the cell fusion was close to 100%, the cell scratch was performed. After replacing the used culture medium with DMEM without FBS, the distance between the scratch area at 0 h was recorded by an Olympus microscope and noted as D0. After 48 hours, the distance of the scratch area was taken from the same position and recorded as Dk48. Repeat this assay three times and cell scratch migration rate = (D0 − Dk48)/D0 × 100%.

Cell migration was tested by Transwell migration assay. The cells were prepared into single-cell suspension by FBS-free medium (HONE1: 1.5 × 105/ml; 5-8F: 2.5 × 105/ml) and were plated in the upper chamber. The lower chamber was added by complete medium which contains 10% FBS. After culture for 24 h, cells that migrated to the lower chamber were fixed and stained by 4% paraformaldehyde and 0.1% crystal violet for 30 min independently. The number of cells that passed to the lower chamber was photographed by positive fluorescence phase contrast microscope (Olympus, Japan).

The Transwell invasion assay was consistent with the migration assay, but the difference is that the upper chamber of the Transwell invasion assay was covered with Matrigel matrix; the ratio of complete medium without FBS and matrix glue was 7.5 : 1.

### 2.9. Statistical Analysis

Statistical Product and Service Solutions (SPSS, Chicago, USA) was utilized for statistical analysis, and *p* < 0.05 (two tails) was considered statistically significant. Measurement data were expressed by mean ± standard deviation. Students *t*-test was used to compare the difference between two groups. ANOVA analysis was utilized when compared with more than two groups. For comparisons between rates, Chi-square test or Fisher exact probability method were used.

## 3. Results

### 3.1. The Expression of SDF2L1 in NPC Tissues and NPC Cell Lines

**Figure 1:**
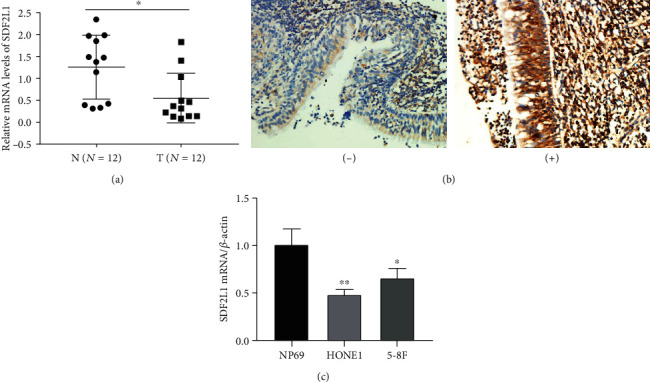
The expression of SDF2L1 in NPC patients. The relative mRNA expression level in NPC patient tissues (a). SDF2L1 expression in NPC and noncancerous nasopharyngeal tissues based on IHC (b). (-) present as negative and (+) present as positive. Relative mRNA expression level in different groups of NPC cells (c). (^∗^*p* = 0.05, ^∗∗^*p* = 0.01).

As shown in Table 1 and Figure 1(a), the mRNA level of SDF2L1 in NPC tissues was 0.549 ± 0.568 while in chronic nasopharyngitis tissues was 1.254 ± 0.729. Consistently, the tissues of ninety-seven patients with NPC and 58 chronic nasopharyngitis patients were applied in the immunohistochemistry assay and it revealed that the positive rate of SDF2L1 in NPC tissues was 2.1% (2/97), which was significantly lower than in chronic nasopharyngitis tissues (50% (29/58)), with all *p* < 0.05 (Table 2 and Figure 1(b)). The relative expression of SDF2L1 in normal nasopharyngeal epithelial cell line NP69 and NPC cell lines HONE1 and 5-8F was detected by qRT-PCR. According to the qRT-RCR, the mRNA levels of SDF2L1 in NP69, HONE1, and 5-8F were 1.009 ± 0.164, 0.487 ± 0.050, and 0.661 ± 0.100, respectively. Interestingly, the mRNA levels of HONE1 and 5-8F cells were statistically significantly lower than that in NP69 cells with *p* = 0.006 and 0.035 (Figure 1(c)).

### 3.2. The Successful Cell Transfection of SDF2L1

**Figure 2:**
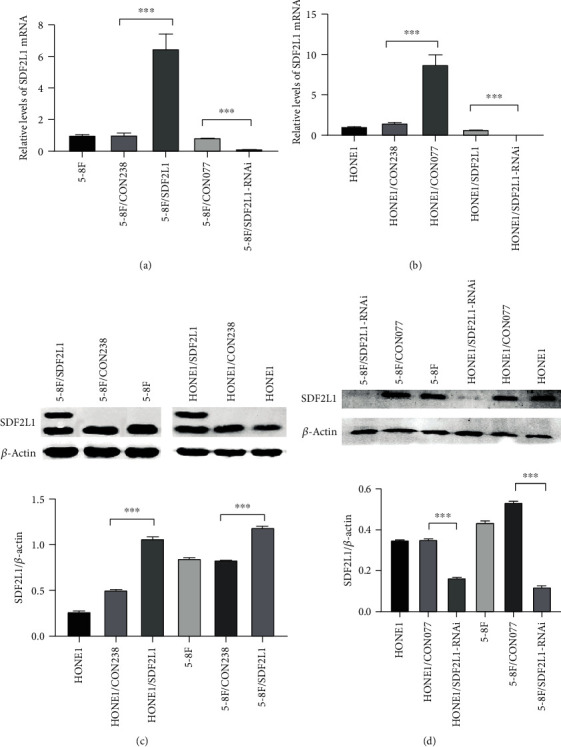
The expression of SDF2L1 in 5-8F and HONE1 cells carrying SDF2L1 transgene with stably SDF2L1 knockdown and overexpression by qRT-PCR (a, b) and Western blot (c, d).

We examined the expression of SDF2L1 in transfected HONE1 and 5-8F cells by using qRT-PCR and Western blotting. These results all indicated that we have established stably transfected HONE1 and 5-8F cells successfully (Figure 2).

### 3.3. SDF2L1 Inhibited NPC Cell Invasion and Migration

**Figure 3:**
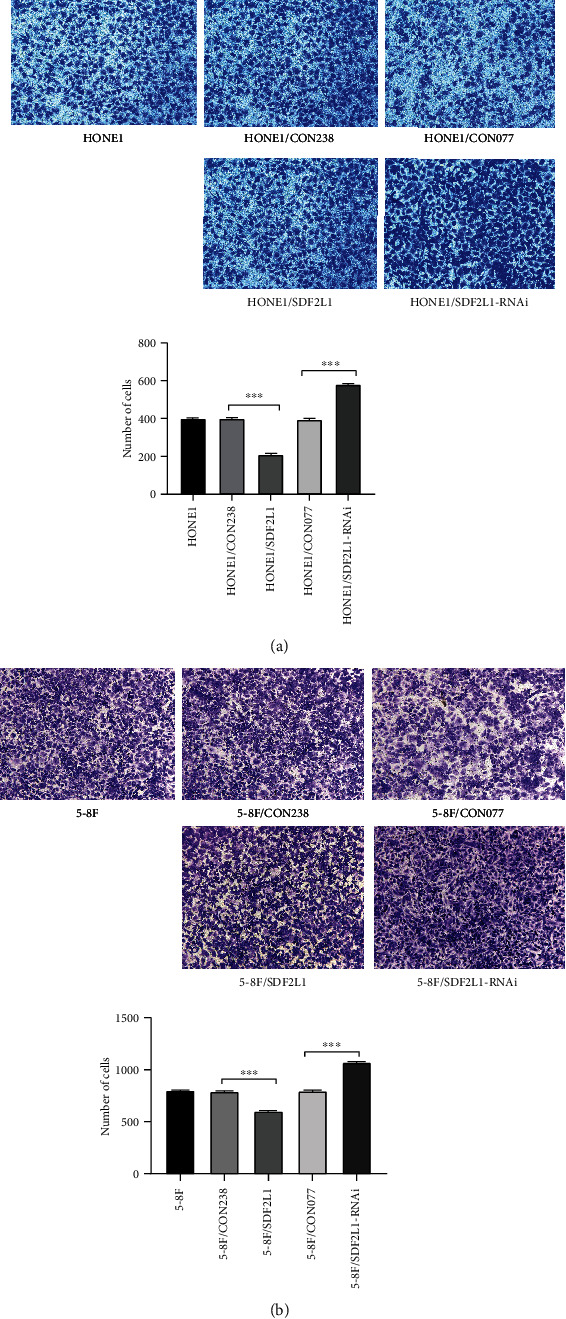
Comparison of the number of transmembrane cells in each group of HONE1 (a) and 5-8F (b) by Transwell migration assays, ^∗∗∗^*p* < 0.001.

Firstly, we applied a scratch migration assay to determine the role of SDF2L1 in NPC cell invasion and migration. We found that when SDF2L1 is overexpressed, the cell scratch migration rates were significantly reduced when compared with the control groups (HONE1/CON238 vs. HONE1/SDF2L1: 52.58 ± 6.14 vs. 40.08 ± 2.71; 5-8F/CON238 vs. 5-8F/SDF2L1: 54.18 ± 2.42 vs. 44.19 ± 3.02; all *p* < 0.05). On the other side, when compared with the control groups, knockdown of SDF2L1 was increased greatly in scratch migration rates (HONE1/CON077 vs. HONE1/SDF2L1-RNAi: 52.07 ± 6.76 vs. 63.58 ± 2.00; 5-8F/CON077 vs. 5-8F/SDF2L1-RNAi: 54.46 ± 3.16 vs. 66.78 ± 1.85; all *p* < 0.05) (Figure 3).

**Figure 4:**
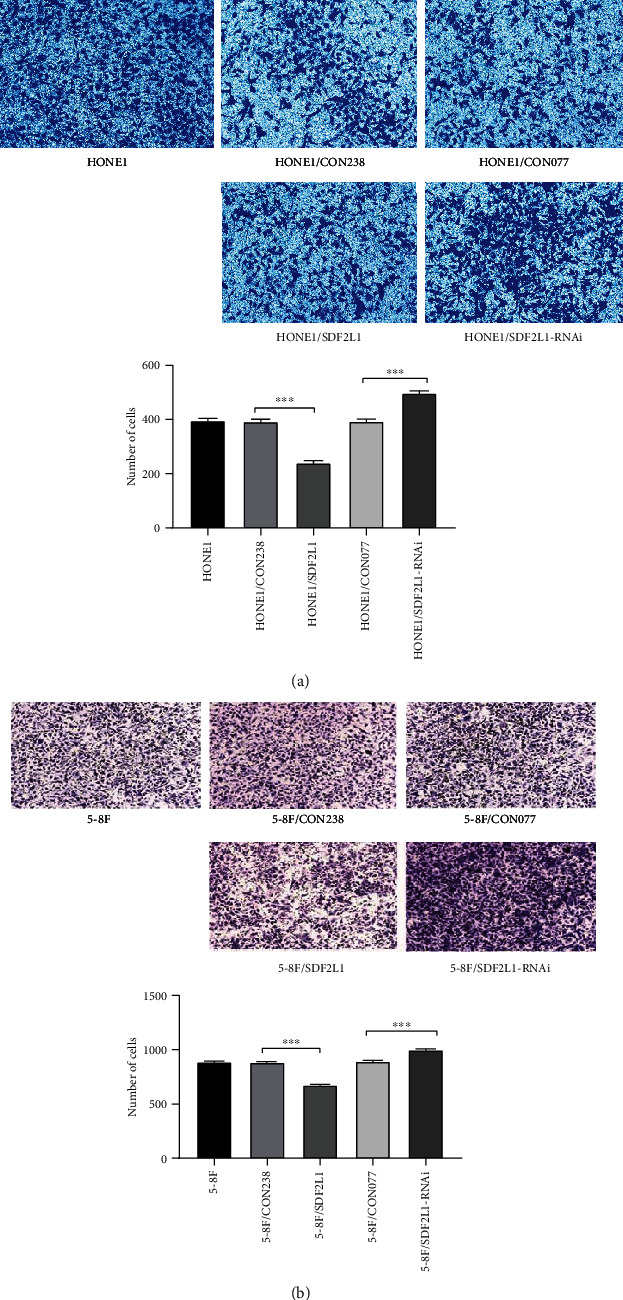
Comparison of the number of transmembrane cells in each group of HONE1 (a) and 5-8F (b) by Transwell invasion assays, ^∗∗∗^*p* < 0.001.

Furthermore, Transwell migration assay revealed that SDF2L1-overexpressing HONE1 (HONE1/CON238 vs. HONE1/SDF2L1: 404.0 ± 11.5 vs. 252.3 ± 10.8) and 5-8F (5-8F/CON238 vs. 5-8F/SDF2L1: 900.3 ± 17.8 vs. 662.3 ± 10.8) cells displayed significantly reduced migration than the control groups (*p* < 0.001). In addition, when the expression of SDF2L1 is knocked down, the migration rates of NPC cells were significantly higher than the control groups (HONE1/CON077 vs. HONE1/SDF2L1-RNAi: 408.0 ± 10.6 vs. 627.3 ± 11.0; 5-8F/CON077 vs. 5-8F/SDF2L1-RNAi: 905.0 ± 13.2 vs. 1093.0 ± 6.2; all *p* < 0.001) (Figure 4).

**Figure 5:**
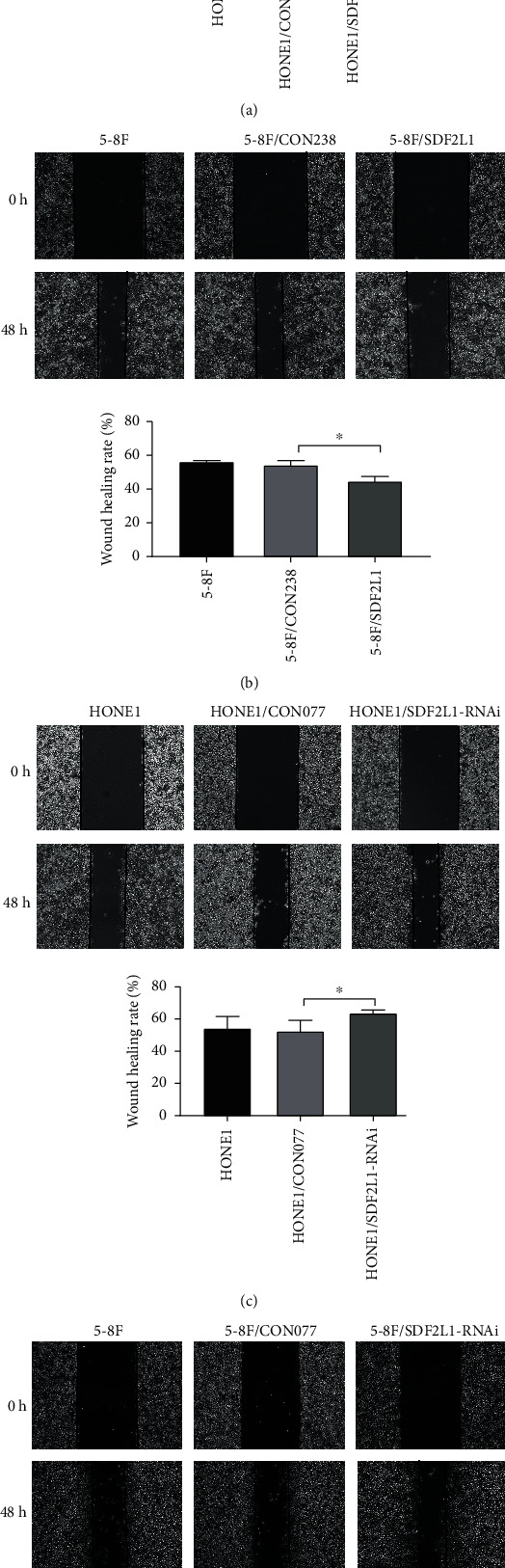
Comparison of relative scratch healing rates of cells in each group of HONE1 and 5-8F after overexpressing SDF2L1 (a, b). Comparison of relative scratch healing rates of cells in each group of HONE1 and 5-8F after silencing SDF2L1 (c, d), ^∗^*p* < 0.05.

Consistently, Transwell invasion assay showed that the cells in the lower chamber of the Transwell were obviously reduced in HONE1/SDF2L1 and 5-8F/SDF2L1 cells compared to the control groups (HONE1/CON238 vs. HONE1/SDF2L1: 392.0 ± 13.9 vs. 239.7 ± 12.7 and 5-8F/CON238 vs. 5-8F/SDF2L1: 884.3 ± 12.1 vs. 674.3 ± 12.9, all *p* < 0.001). Beyond that, the SDF2L1-knockdown HONE1 and 5-8F cells showed the opposite results (HONE1/CON077 vs. HONE1/SDF2L1-RNAi: 393.3 ± 15.3 vs. 497.0 ± 20.2 and 5-8F/CON077 vs. 5-8F/SDF2L1-RNAi: 894.3 ± 14.0 vs. 999.3 ± 18.0; all *p* < 0.001) (Figure 5).

All of these results revealed that SDF2L1 may play an inhibition role in the invasion and migration of nasopharyngeal carcinoma.

### 3.4. SDF2L1 Inhibited NPC Cell Proliferation

**Figure 6:**
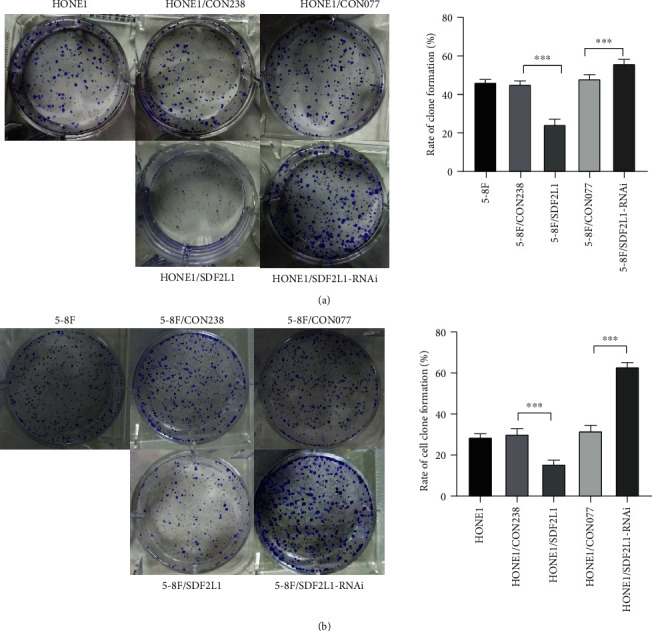
Comparison of the clonal formation ability of cells in each group. HONE1 (a). 5-8F (b). ^∗∗∗^*p* < 0.001.

**Figure 7:**
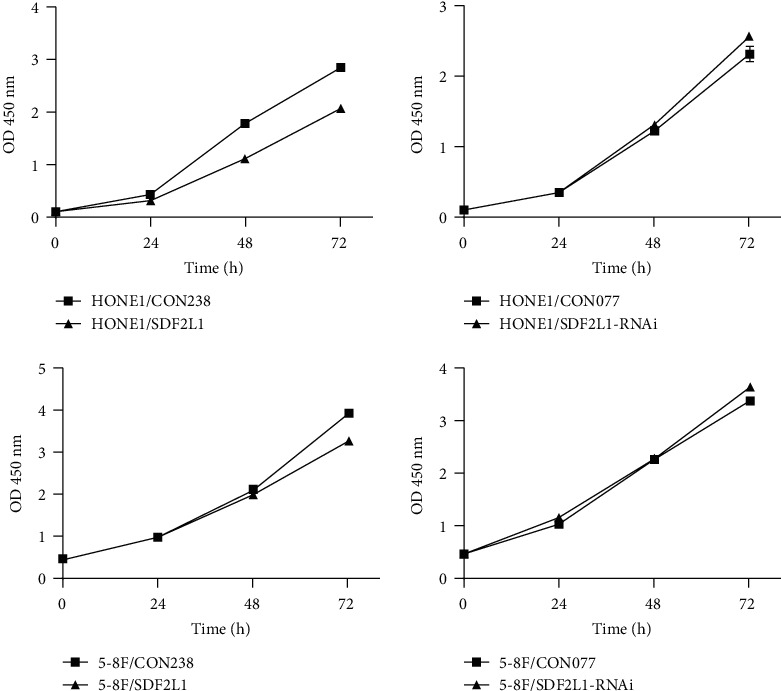
Comparison of CCK-8 OD450 in each group at each time point.

We then investigated the value of SDF2L1 on cell proliferation in vitro using cell clone formation and CCK-8 assay. These revealed that the stably transfected HONE1 and 5-8F cells overexpressing SDF2L1 inhibited cell proliferation, when compared with the control groups. Consistently, the knockdown of SDF2L1 significantly promoted NPC cell proliferation in vitro (Figures 6 and 7).

## 4. Discussion

The activation of oncogenes and deletion of cancer suppressor genes are considered to be important factors in the development of NPC [8–10]. In recent years, a number of literatures have reported that the expression levels of tumor suppressor genes were related to the occurrence and development of NPC. Gao et al. found that the activation of oncogene LASP1 can downregulate the expression of antioncogene PTEN, which could promote the proliferation, invasion, and metastasis of NPC cells [9]. The low expression of SDF2L1 has been observed in breast and ovarian cancer. Kang et al. reported that the low expression of SDF2L1 was correlated with prognosis of breast cancer patients [11]. Furthermore, a meta-analysis found that the expression levels of SDF2L1 were correlated with poor prognosis of ovarian cancer patients [12]. Therefore, we speculated that SDF2L1 might be a potential tumor suppressor gene in NPC and may be involved in the occurrence and development of NPC.

Compared with chronic nasopharyngitis tissues, we found that the mRNA and protein levels in NPC tissues were significantly lower. It suggested that SDF2L1 may play an important role in the occurrence and development of NPC. In line with tissue assay, we found that the relative expression of SDF2L1 in NPC cell lines (HONE1 and 5-8F) were significantly lower than that in normal cell lines (NP69). All of these suggested that the downexpression of SDF2L1 may play an important role in the occurrence and development of NPC.

The ability of cell migration and invasion are closely related to the occurrence and development of tumor and strong metastasis and invasiveness are common characteristics of malignant tumors [13]. NPC can metastasize to a variety of organs, such as the spleens, livers, lungs, and bone, which greatly increases the difficulty in the treatment of NPC [14–16]. A range from 17% to 54% NPC patients misses the opportunity for treatment due to distant metastasis [17, 18]. But until now, the pathogenesis of distant metastasis in NPC has not been fully understood. Interestingly, we found that overexpression of SDF2L1 inhibited NPC cell migration and invasion and silencing the expression of SDF2L1 has the opposite effect. These results indicated that SDF2L1 might be important for protective behaviors of NPC patients.

Cancer is characterized by the rapid development of abnormal cells that divide uncontrollably and have the ability to destroy body tissues. In the context of cell proliferation rapidly, there is a great demand for protein, and the endoplasmic reticulum is the site of protein synthesis [19]. Previous studies have shown that SDF2L1 is closely related to endoplasmic reticulum stress which is involved in the occurrence and development of a variety of diseases, especially cancers [6, 20–23]. According to the cell clone formation and CCK-8 assay, overexpression of SDF2L1 significantly decreased the colony formation and growth of HONE1 and 5-8F cells and downexpression of SDF2L1 has the opposite results. These results revealed that SDF2L1 can inhibit cell proliferation in NPC cells.

In conclusion, our study demonstrated that SDF2L1 play a crucial role in the occurrence and development of NPC. SDF2L1, as a factor related to endoplasmic reticulum stress, may be a new therapeutic target or prognostic indicator for NPC. The exact mechanism of endoplasmic reticulum stress and NPC remains to be further studied.

## Figures and Tables

**Table 1:** The relative expression level of SDF2L1 mRNA in nasopharyngitis tissues (N) and nasopharyngeal carcinoma tissues (T).

Group	*n*	2^−ΔΔCt^ (^−^*x* ± *s*)	*p* value
N	12	1.254 ± 0.729	0.015
T	12	0.549 ± 0.568	

**Table 2:** The expression of SDF2L1 was detected by immunostaining in NPC tissues (T) and nasopharyngitis tissues (N).

	n	The expression of SDF2L1		Positive rate (%)	*χ* ^2^	*p* value
		Negative	Positive			
N	58	5	53	91.4	72.942	<0.001
T	97	77	20	20.6		

## Data Availability

The results in this study are available from the corresponding author on reasonable request.
