# Corrigendum to “SDF2L1 Inhibits Cell Proliferation, Migration, and Invasion in Nasopharyngeal Carcinoma”

**DOI:** 10.1155/2022/9898479

**Published:** 2022-05-12

**Authors:** Liqian Zhang, Zunni Zhang, Liuqun Qin, Xiang Shi, Qisheng Su, Wuning Mo

**Affiliations:** Department of Clinical Laboratory, First Affiliated Hospital of Guangxi Medical University, Nanning, Guangxi Zhuang Autonomous Region, China

In the article titled “SDF2L1 Inhibits Cell Proliferation, Migration, and Invasion in Nasopharyngeal Carcinoma” [1], the authors wish to correct Figures 3(a) and 3(b) as HONE1, HONE1/CON238, and HONE1/SDF2L1 were identified as being duplicated. The authors explained that this was due to an error made when uploading the manuscript and the raw data has been provided upon request. The corrected image is shown below:

## Figures and Tables

**Figure 1:**
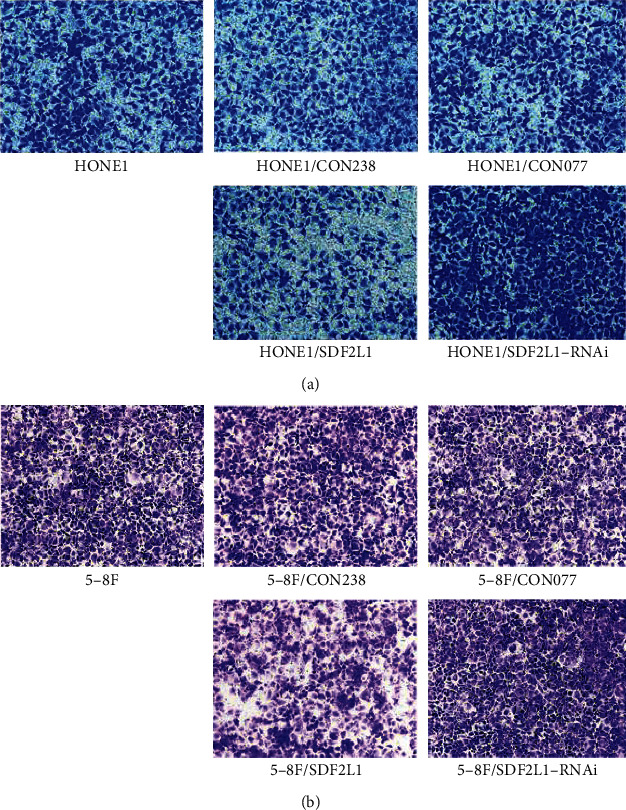
Comparison of the number of transmembrane cells in each group of HONE1 (a) and 5-8F (b) by Transwell migration assays, ^∗∗∗^*p* < 0.001.
